# Exploring neurometabolic alterations in bipolar disorder with suicidal ideation based on proton magnetic resonance spectroscopy and machine learning technology

**DOI:** 10.3389/fnins.2022.944585

**Published:** 2022-09-09

**Authors:** Jiayue Chen, Xinxin Zhang, Yuan Qu, Yanmin Peng, Yingchao Song, Chuanjun Zhuo, Shaohong Zou, Hongjun Tian

**Affiliations:** ^1^Department of Psychiatry, Tianjin Fourth Center Hospital, The Fourth Central Clinical College, Tianjin Medical University, Tianjin, China; ^2^Department of Key Laboratory of Real Time Imaging of Brian Circuits in Psychiatry and Neurology (RTIBNP_Lab), Tianjin Fourth Center Hospital, The Fourth Central Clinical College, Tianjin Medical University, Tianjin, China; ^3^Department of Psychiatry, School of Basic Medical Science, Tianjin Medical University, Tianjin, China; ^4^Department of Medical Imaging, Tianjin Children's Hospital, Tianjin, China; ^5^Department of Radiology, People's Hospital of Xinjiang Uygur Autonomous Region, Ürümqi, China; ^6^School of Medical Imaging, Tianjin Medical University, Tianjin, China; ^7^Tianjin Key Laboratory of Functional Imaging, Tianjin Medical University, Tianjin, China; ^8^Psychiatric-Neuroimaging-Genetics and Comorbidity Laboratory (PNGC_Lab), Tianjin Anding Hospital, Mental Health Teaching Hospital of Tianjin Medical University, Tianjin, China; ^9^Department of Psychiatry, First Affiliated Hospital of Zhengzhou University, Zhengzhou, China; ^10^Department of Psychiatry, First Hospital/First Clinical Medical College of Shanxi Medical University, Taiyuan, China; ^11^Department of Clinical Psychology, People's Hospital of Xinjiang Uygur Autonomous Region, Ürümqi, China

**Keywords:** bipolar disorder, suicidal ideation, proton magnetic resonance spectroscopy, machine learning, multivariate pattern analysis, support vector machine

## Abstract

Bipolar disorder (BD) is associated with a high risk of suicide. We used proton magnetic resonance spectroscopy (^1^H-MRS) to detect biochemical metabolite ratios in the bilateral prefrontal white matter (PWM) and hippocampus in 32 BD patients with suicidal ideation (SI) and 18 BD patients without SI, identified potential brain biochemical differences and used abnormal metabolite ratios to predict the severity of suicide risk based on the support vector machine (SVM) algorithm. Furthermore, we analyzed the correlations between biochemical metabolites and clinical variables in BD patients with SI. There were three main findings: (1) the highest classification accuracy of 88% and an area under the curve of 0.9 were achieved in distinguishing BD patients with and without SI, with N-acetyl aspartate (NAA)/creatine (Cr), myo-inositol (mI)/Cr values in the bilateral PWM, NAA/Cr and choline (Cho)/Cr values in the left hippocampus, and Cho/Cr values in the right hippocampus being the features contributing the most; (2) the above seven features could be used to predict Self-rating Idea of Suicide Scale scores (r = 0.4261, *p* = 0.0302); and (3) the level of neuronal function in the left hippocampus may be related to the duration of illness, the level of membrane phospholipid catabolism in the left hippocampus may be related to the severity of depression, and the level of inositol metabolism in the left PWM may be related to the age of onset in BD patients with SI. Our results showed that the combination of multiple brain biochemical metabolites could better predict the risk and severity of suicide in patients with BD and that there was a significant correlation between biochemical metabolic values and clinical variables in BD patients with SI.

## Introduction

Bipolar disorder (BD) is a lifelong severe mental disorder characterized by alternating high and low emotions. Approximately 2%−3% of individuals in the world are troubled by bipolar and related disorders. The World Health Organization (WHO) reported that BD is expected to rise to sixth place on the list of global burdens of disease by 2030. Some studies have shown that BD is the disease associated with the highest risk of suicide among all major mental disorders (Merikangas et al., 2011), and the associated suicide rate is 20 to 30 times higher than that in the common population (Plans et al., 2019; Carvalho et al., 2020). In terms of suicide risk, suicidal ideation (SI) is considered to be one of the important predictors of future suicidal behavior (Nock et al., 2008). Therefore, paying close attention to SI can reduce the risk of early suicide in patients with BD. However, the current assessment of suicide risk is still mainly based on many sociodemographic characteristics and clinical risk factors, which usually have poor predictive accuracy. In addition, due to the influence of patients' subjectivity and shame, almost 80% of patients who attempted suicide do not explain their suicidal thoughts to doctors (Gosnell et al., 2019). Consequently, strengthening the search for specific neurobiological markers will help to predict future suicide risk in BD patients with SI.

At present, the neurobiological mechanisms underlying SI in patients with BD are unknown. Magnetic resonance imaging (MRI) technology has been widely used to understand the pathophysiological mechanisms underlying mental diseases, and such research has made corresponding progress in revealing the neurobiological changes in brain regions of suicidal patients with BD. For example, the graph theory analysis of global brain functional connectivity in resting-state functional MRI showed that the distribution of intrinsic connectivity in the bilateral ventromedial prefrontal cortex of patients with BD who attempted suicide was significantly lower than that in patients without attempted suicide and was related to the severity of SI (Sankar et al., 2022). Relevant studies have found that in patients with a current or prior diagnosis of depression or BD, the intensity of SI was associated with weaker connections of the limbic network with the hippocampus, default mode network, dorsal attention network, and executive control network (Chin Fatt et al., 2021). In studies evaluating low-frequency fluctuations (ALFF) and gray matter volume in the prefrontal cortex, it was shown that the ALFF values in the medial prefrontal cortex, ventral prefrontal cortex, and dorsolateral prefrontal cortex in BD patients with suicide attempts were significantly higher than those in patients without suicide attempts (Zhao et al., 2021). In gray matter and white matter-related studies, bilateral hippocampal gray matter volume and right ventral frontal white matter fractional anisotropy were found to decrease in BD patients with suicide attempts (Fan et al., 2019). From the above research, we can speculate that the structural and functional abnormalities in the prefrontal lobe and hippocampus may be related to SI in patients with BD.

Proton magnetic resonance spectroscopy (^1^H-MRS) is a non-invasive and non-radioactive technique used to study the levels of biochemical metabolites in the brain, including N-acetyl aspartate (NAA), choline (Cho), myo-inositol (mI), and creatine (Cr), which can provide relevant information about neuronal integrity and neurotransmitter levels. Some studies have shown that the NAA/Cr values in the left prefrontal white matter (PWM) in patients with BD II (Zhong et al., 2014) and bilateral PWM in depressed patients with BD, compared with healthy controls, were decreased (Lai et al., 2019), furthermore, NAA/Cr+phosphocreatine and NAA/Cho values in the bilateral hippocampus were significantly decreased in patients with first-episode BD I (Atmaca et al., 2006). Glutamic acid and glutamine complex (Glx) levels and NAA/Glx in the anterior cingulate cortex could distinguish depression patients with and without SI (Lewis et al., 2020). However, there are few studies on ^1^H-MRS in BD patients with suicide, and the results have either not been significant or not been very consistent (Rocha et al., 2015; Zhong et al., 2018).

In recent years, multivariate pattern analysis (MVPA) has become an effective analysis method that can often detect differences in neuroimaging data that cannot be detected by traditional univariate statistical methods by combining information from many features (Nielsen et al., 2020). Support vector machine (SVM) has been one of the most widely used machine learning algorithms to identify neurobiological markers of various neuropsychiatric disorders, which has high sensitivity and specificity in distinguishing patients with BD from those with other neuropsychiatric disorders or healthy subjects, as well as predicting clinical outcomes of neuropsychiatric disorders (Orrù et al., 2012; Liu et al., 2015, 2017a; Librenza-Garcia et al., 2017; Orban et al., 2018; Schwarz et al., 2019; Yang et al., 2019; Ji et al., 2020; Li et al., 2020). However, to date, the research on ^1^H-MRS of BD using the SVM algorithm is very limited, and only one study described the potential of ^1^H-MRS in predicting the first emotional episode of young BD offspring by using SVM (Zhang et al., 2021). Research on ^1^H-MRS in BD patients with SI is nonexistent.

Therefore, in this study, we used ^1^H-MRS to detect the biochemical metabolite ratios in the bilateral PWM and hippocampus in BD patients with and without SI, identified potential brain biochemical differences in BD patients with SI, and used these abnormal metabolite ratios to predict the severity of suicide risk based on the SVM algorithm. In addition, we examined the relationships between biochemical metabolite ratios and clinical variables. In short, the purpose of this study was to explore changes in brain biochemical metabolites in BD patients with SI, to identify high-accuracy neuroimaging predictors that may be used to evaluate the risk of suicide, and then to provide early identification and assessment of the risk or tendency of suicidal behavior in patients with BD in future.

## Methods

### Participants and clinical assessments

We recruited 50 patients with BD from the Department of Clinical Psychology of the People's Hospital of Xinjiang Uygur Autonomous Region in China. There was a restriction on the age of all participants, who ranged from 18 to 55 years. The diagnosis of BD was confirmed by two experienced psychiatrists according to the structured clinical interview criteria of the Diagnostic and Statistical Manual of Mental Disorders, Fourth Edition, Text Revision (DSM-IV-TR). The evaluation of the clinical status was conducted utilizing the 17-item Hamilton Depression Scale (HAMD) (Hamilton, 1960) and the Young Mania Rating Scale (YMRS) (Young et al., 1978). All patients had a YMRS total score <7 and a 17-item HAMD total score >17. Moreover, an inclusion criterion for all participants was that they were right-handed. The exclusion criteria included the following: (1) other mental disorders meeting DSM-IV-TR criteria; (2) a history of organic brain disorder and serious physical illness; (3) a history of epilepsy, severe brain injury, or coma caused by other reasons for more than 5 min; (4) alcohol or drug abuse; (5) pregnancy or lactation; and (6) contraindications for magnetic resonance scanning, such as metal implants or claustrophobia.

We used the Self-rating Idea of Suicide Scale (SIOSS) to assess whether the patients had SI. There were 26 items on the SIOSS that included four factors: despair factor, optimism factor, sleep factor, and concealment factor. All items were scored with a “yes” or “no” answer. The assessment criteria for SI were the sum of the total scores of the despair factor, optimism factor, and sleep factor ≥12 and the concealment score <4. The higher the score was, the stronger the SI. The scale is simple and easy for patients to understand, and it has good validity and reliability (Xia et al., 2002). According to SIOSS scores, there were 32 patients with SI and 18 patients without SI. In addition, our study evaluates the severity of anxiety symptoms in all patients with BD by utilizing the 14-item Hamilton Anxiety Scale (HAMA).

### Image data acquisition

The MRI scanning was conducted on all subjects using an Ingenia 3.0T MRI scanner (Ingenia, Philips Healthcare, Netherlands) with a 15-channel phased-array head coil. The following precautions were taken before scanning: (1) coffee, tobacco, and alcohol were banned in 24 h before the MRI scanning, and the body temperature need to be kept at a normal level to rule out feverish patients; (2) the doctor told the subjects not to move during the MRI scanning; and (3) the subjects were required to rest quietly for half an hour before the examination. In addition, earplugs and headphones were used to reduce noise, and head movement was reduced by using foam pads.

One 3DT1FSPGR sequence that covered the whole brain was used for the anatomical localization of MRS. T2-weighted images (T2WI) [repetition time (TR) = 3,000 ms, echo time (TE) = 95 ms], T1-weighted images (T1W-IR) [TR = 2,000 ms, TE = 20 ms, inversion time (TI) = 800 ms], and T2 fluid-attenuated inversion recovery (T2FLAIR) (TR = 8,000 ms, TE = 270 ms, TI = 2,000 ms) were routinely conducted. The voxel size = 0.8 × 0.8 × 6 mm^3^, there were 18 slices, with a field of view (fov)=24 cm. The scanning planes of three sequences were duplicated by each other. Before ^1^H-MRS sequence localization, it was necessary to judge whether there were organic lesions, and the anatomical positions of the bilateral PWM and hippocampus were determined by experienced radiologists. In three dimensions, none of the volume of interest (VOI) involved sulci or cerebrospinal fluid. The size of a single voxel was 20 × 20 × 20 mm^3^. The scanning parameters were as follows: TE = 35 ms; TR = 2,000 ms; and average (superposition) number of signals (NSA) = 96. To prevent the disturbance of spectral line quality by some disadvantageous factors surrounding the spectrum voxel, the saturation band could be manually placed near the VOI, which was essential to ensure that the air, bone, fat, or blood vessels surrounding the VOI were adequately suppressed. During scanning, the chemical shift selective saturation method was used to optimize the water suppression to ensure that the water suppression rate was >99% and the full width at half maximum of the water peak was <10 Hz. The acquisition time of the ^1^H-MRS sequence was 10 min (Chen et al., 2021).

### Data processing

The spectral view software of the Philips 3.0T workstation (ISP 7.0, Philips Healthcare, the Netherlands) was used for ^1^H-MRS data postprocessing. The specific data processing steps were as follows: residual water peak removal, signal attenuation correction, spectral line interpolation, Fourier transform, spectral line phase correction, baseline level adjustment, the selection of peak frequency position, line width setting, and peak Gaussian fitting. The NAA/Cr, Cho/Cr, and mI/Cr ratios in the bilateral PWM and hippocampus were used to analyze the changes in brain biochemical metabolism, and an experienced radiologist evaluated the quality of the spectrum and analyzed the data.

### Univariate statistical analysis

The univariate data were analyzed by SPSS 24.0 software, and the significance level of the two tails was set at *p* < 0.05. When the data were continuous variables that conformed to a normal distribution, the *t-*test was conducted, and the data are expressed as the mean ± standard deviation. When the continuous variable did not conform to a normal distribution, the non-parametric test was selected, and the data are expressed as the median (upper quartile, lower quartile). The data of discontinuous variables were analyzed by the chi-square test. Then, the results with statistically significant differences between the two groups were further corrected by the Bonferroni correction method for multiple comparisons. In determining whether the concentration of brain biochemical metabolites in BD patients with SI was related to clinical variables, Pearson correlation analysis was used for the two groups of variables conforming to the normal distribution, and Spearman correlation analysis was used for those not conforming to the normal distribution. In addition, we plotted the receiver operating characteristic (ROC) curves and calculated corresponding areas under the curve (AUC) for the abnormal metabolic values of BD patients with and without SI.

### MVPA

This analysis was conducted using SVM (i.e., support vector classification, SVC) from the MVPANI toolbox (Peng et al., 2020). We first used the leave-one-out cross-validation (LOOCV) method, leaving only one subject for the test set each time, and the remaining 49 subjects for the training set, which resulted in the model being trained 50 times and tested 50 times. Although the calculation method using LOOCV is complicated, it has a high utilization rate of samples, which is suitable for small sample research. Each feature was standardized before cross-validation: the values in each row (each row represents a sample) were normalized by transforming all values in each sample to z scores with a mean of 0 and a standard deviation (SD) of 1 using the following equation:


$$\begin{array}{l} z_{ij}=\frac{x_{ij}-mean\left(x_{i.}\right)}{SD\left(x_{i.}\right)}for\;the\;i^{th}\;row \end{array}$$

We adopted the feature selection method of F-score in this study. The specific process was shown as follows: in the process of each LOOCV, all features were arranged in order from high to low according to the size of F-values of an F-test in BD patients with and without SI in the training dataset. Then, we selected the number of the feature with the highest F-values of 10% (the number of the feature was one) to train a new classifier in the training dataset and the performance of the classifier was tested using the reserved test dataset, so the classification accuracy of this LOOCV was generated. According to the LOOCV process, 50 models and 50 feature sets were generated, and the features contained in each feature set in the 50 feature sets were not all the same. In addition, the features in each feature set have corresponding weight values. Finally, we could calculate the average classification accuracy, the average weight values of the features, and the frequency of each feature of 50 models. Next, increase the number of feature by 10% each time, and repeat the above feature selection process until all features were selected, and the number of features was 2, 3, 4, 6, 7, 8, 9, 10, 12. Therefore, 10 feature sets with different feature numbers and 10 average classification accuracies were finally obtained. The statistical significance of classification accuracy was determined by the permutation test, and the significance threshold was *p* < 0.05. In this study, we conducted 10,000 random permutation tests, obtained 10,000 random classification accuracies, and then used these 10,000 random classification accuracies to construct the null distribution. The *p-*value was the percentage greater than or equal to the actual classification accuracy (*p* = 0.0001, i.e., 1/10,000). In the training set of feature selection with the F-score, because we performed 10 independent MVPA analyses, the above *p*-values calculated from the permutation tests needed to be further corrected by the Bonferroni correction method for multiple comparisons. In addition to obtaining classification accuracy, we calculated the receiver operating characteristic (ROC) curves and the corresponding areas under the curve (AUCs) for each classification.

Furthermore, SVM (i.e., support vector regression, SVR) was applied to predict SIOSS scores in patients with BD. We used the biochemical metabolic values corresponding to the highest classification accuracy from the above feature selection process as the feature and SIOSS scores in patients with BD as the regression target of the regression analysis. We also made use of the LOOCV method to divide all subjects into the training set and test set, and each feature was standardized in the process of cross-validation (the method was the same as before). Finally, we calculated the Pearson correlation coefficient between predicted SIOSS scores and observed SIOSS scores. Similarly, we used the permutation test to determine the statistical significance of the Pearson correlation coefficient using the followed specific methods. The predicted SIOSS scores of patients with BD were randomly disrupted. With the disrupted SIOSS scores, we recalculated the Pearson correlation coefficients between the predicted SIOSS scores and the observed SIOSS scores. We repeated the above process 10,000 times and compared the actual Pearson correlation coefficient with the null distribution based on the null distribution composed of 10,000 Pearson correlation coefficients. The *p-*value was the proportion of the Pearson correlation coefficient obtained by random permutation tests greater than or equal to the actual value in 10,000 random permutation tests. The level of statistical significance was *p* < 0.05.

## Results

### Demographics

Thirty-two BD patients with SI (11 men, 21 women; 34.69 ± 10.51 years old; age range: 18–54 years) and 18 BD patients without SI (nine men, nine women; 32.89 ± 10.97 years old; the age range: 18–54 years) underwent demographics and clinical evaluation in this study. Table 1 shows the demographic and clinical variables of all participants in the study. There were no significant differences in age, gender, educational level, family history, duration of illness, and age of onset between BD patients with and without SI (all *p* > 0.05). In terms of clinical symptoms, the SIOSS score in BD patients with SI was higher than that in BD patients without SI (*p* < 0.05). No significant differences were found in the 17-item HAMD and 14-item HAMA scores between the groups (all *p* > 0.05).

**Table 1:** Demographic and clinical data of BD patients with and without SI.

	**BD with SI (*n* = 32)**	**BD without SI (*n* = 18)**	**z/t/χ2**	***p***
Gender (male/female)	11/21	9/9	1.172	0.279
Positive family history (yes/no)	8/24	1/17	1.781	0.182
Education (years)	16 (15, 16)	16 (15, 16)	−0.494	0.621^b^
Duration of illness (month)	48 (24, 81)	60 (20.5, 63)	−0.457	0.647^b^
Age (years)	34.69 ± 10.51	32.89 ± 10.97	0.572	0.570^a^
Age of onset (years)	29.88 ± 10.11	28.61 ± 9.64	0.432	0.668^a^
17-item HAMD score	18.5 (18, 21)	19 (18, 19.25)	−0.492	0.623^b^
14-item HAMA score	13.97 ± 4.04	14.67 ± 3.80	−0.598	0.553^a^
SIOSS score	18 (15, 20)	10.5 (6, 11)	−5.856	0.000^b, *^

BD, bipolar disorder; SI, suicidal ideation; HAMD, Hamilton Depression Scale; HAMA, Hamilton Anxiety Scale; SIOSS, Self-rating Idea of Suicide Scale; ^a^Independent samples t-test; ^b^Mann–Whitney U test; ^*^p < 0.05 significant.

### Comparisons of ^1^H-MRS in the bilateral PWM and hippocampus of BD patients with and without SI

**Figure 1:**
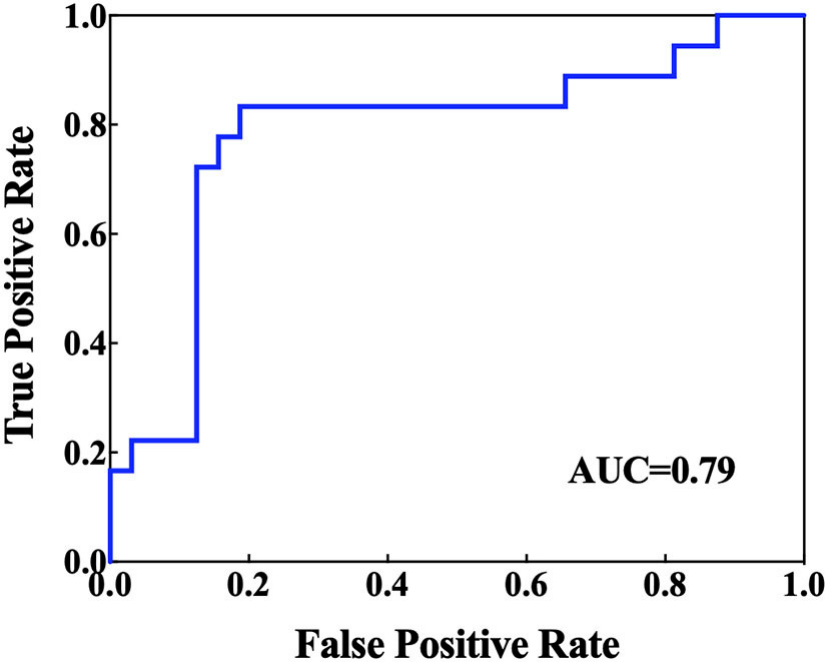
Receiver operating characteristic (ROC) curves and corresponding areas under the curve (AUCs) for the brain biochemical metabolites that showed significant differences in the univariate statistical analysis.

Table 2 presents the comparative results of the NAA/Cr, Cho/Cr, and mI/Cr ratios in the bilateral PWM and hippocampus of BD patients with and without SI. After using the Bonferroni correction method, the Cho/Cr ratios in the left hippocampus were significantly lower in BD patients with SI than in BD patients without SI (*p* < 0.05). However, there were no significant differences in the Cho/Cr ratios in the right hippocampus, NAA/Cr and mI/Cr ratios in the bilateral hippocampus, and NAA/Cr, Cho/Cr, and mI/Cr ratios in the bilateral PWM between the two groups (all *p* > 0.05). Furthermore, the AUC of the Cho/Cr values in the left hippocampus was 0.79 (Figure 1).

**Table 2:** Comparisons of proton magnetic resonance spectroscopy in the bilateral PWM and hippocampus of BD patients with and without SI.

	**BD with SI (*n* = 32)**	**BD without SI (*n* = 18)**	**z/t**	***p***	***p^*c*^***
Left PWM					
NAA/Cr	2.02 (1.78, 2.07)	1.78 (1.37, 2.06)	−1.598	0.110^b^	
Cho/Cr	0.95 ± 0.33	0.98 ± 0.39	−0.247	0.806^a^	
mI/Cr	0.52 (0.41, 0.61)	0.53 (0.45, 1.11)	−1.275	0.202^b^	
Right PWM					
NAA/Cr	1.79 (1.54, 1.91)	1.63 (1.27, 1.71)	−2.024	0.043^b,*^	0.516
Cho/Cr	1.11 (0.77, 1.17)	0.98 (0.69, 1.15)	−0.689	0.491^b^	
mI/Cr	0.97 (0.55, 2.06)	0.5 (0.35, 0.68)	−2.615	0.009^b,*^	0.108
Left hippocampus					
NAA/Cr	1.80 (1.52, 1.88)	2.09 (1.67, 2.31)	−1.827	0.068^b^	
Cho/Cr	0.90 (0.74, 0.90)	1.33 (1.04, 1.44)	−3.369	0.001^b,*^	0.012*
mI/Cr	0.75 (0.61, 0.86)	0.78 (0.47, 0.86)	−0.214	0.830^b^	
Right hippocampus					
NAA/Cr	1.86 (1.55, 1.86)	1.78 (1.40, 1.96)	−0.898	0.369^b^	
Cho/Cr	1.06 (0.78, 1.06)	1.12 (0.78, 1.18)	−1.204	0.229^b^	
mI/Cr	0.71 (0.44, 0.71)	0.65 (0.57, 0.70)	−0.857	0.391^b^	

BD, bipolar disorder; SI, suicidal ideation; PWM, prefrontal whiter matter; NAA, N-acetylaspartate.

Cho, choline; mI, myo-inositol; Cr, creatine; ^a^Independent samples t-test; ^b^Mann–Whitney U test.

^c^Bonferroni correction; ^*^p < 0.05 significant.

### MVPA of biochemical metabolite ratios in BD patients with and without SI

**Figure 2:**
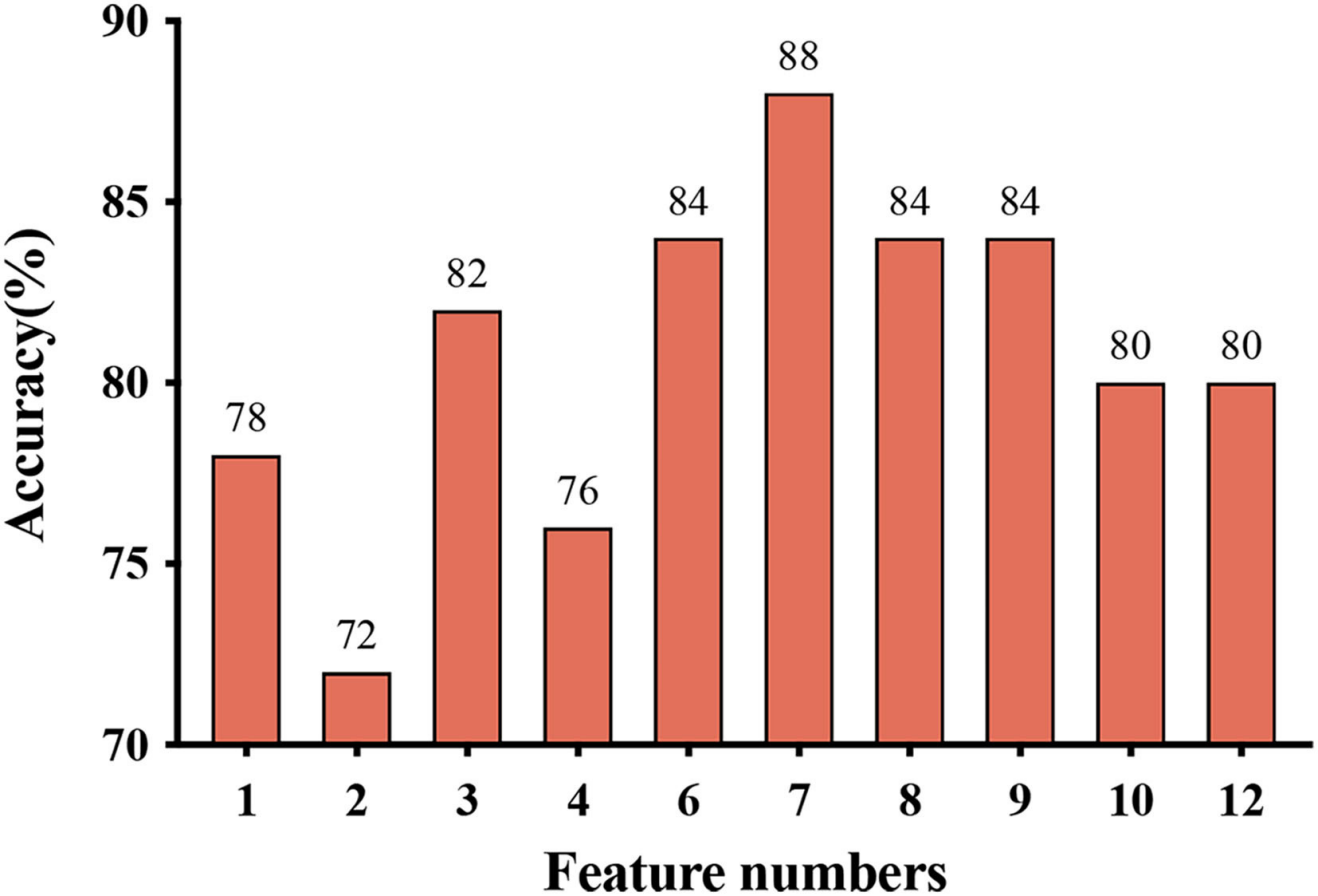
Feature numbers and classification accuracy for each classifier when using feature selection.

**Figure 3:**
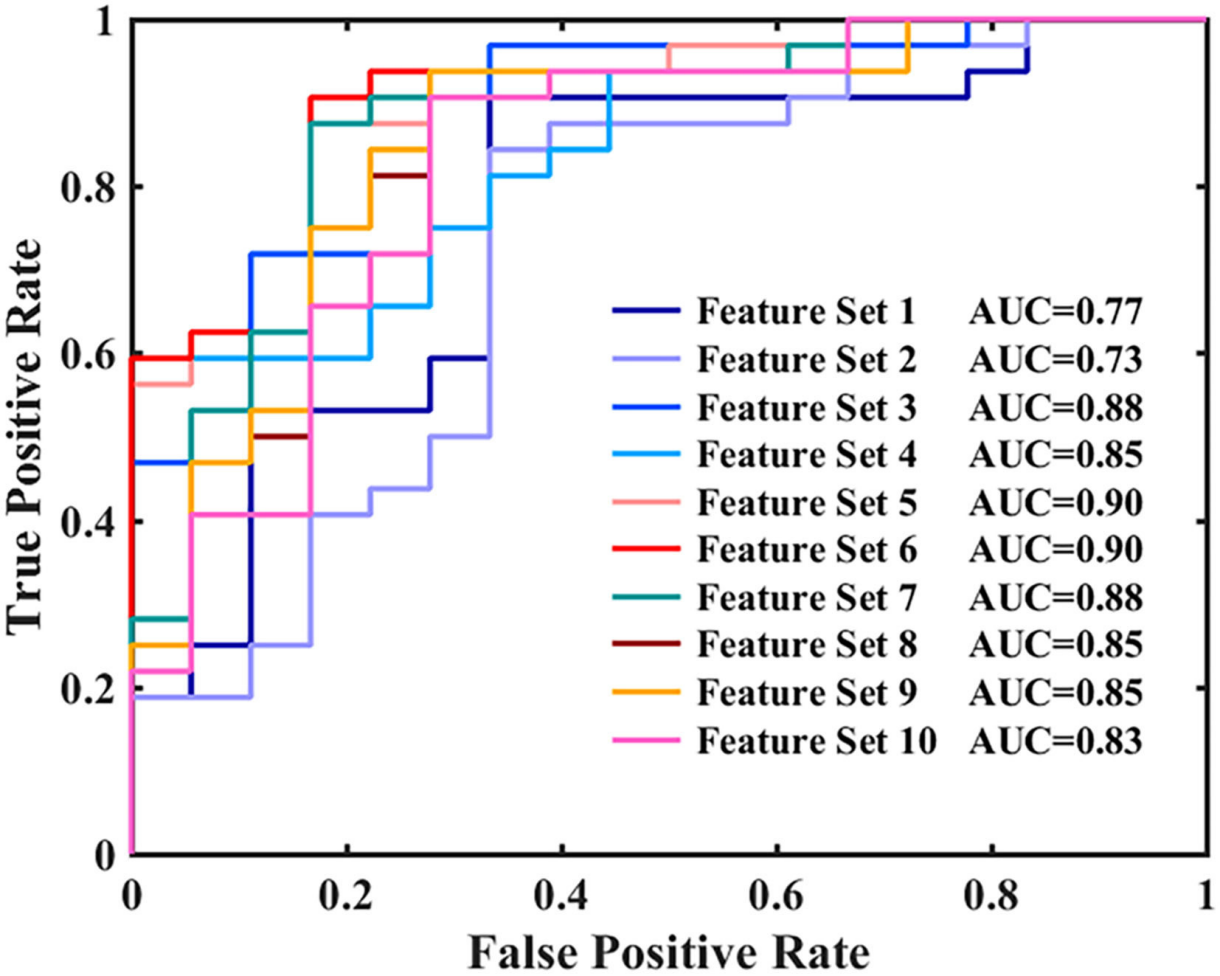
Receiver operating characteristic (ROC) curves and areas under the curves (AUCs) for each classifier when using feature selection.

**Figure 4:**
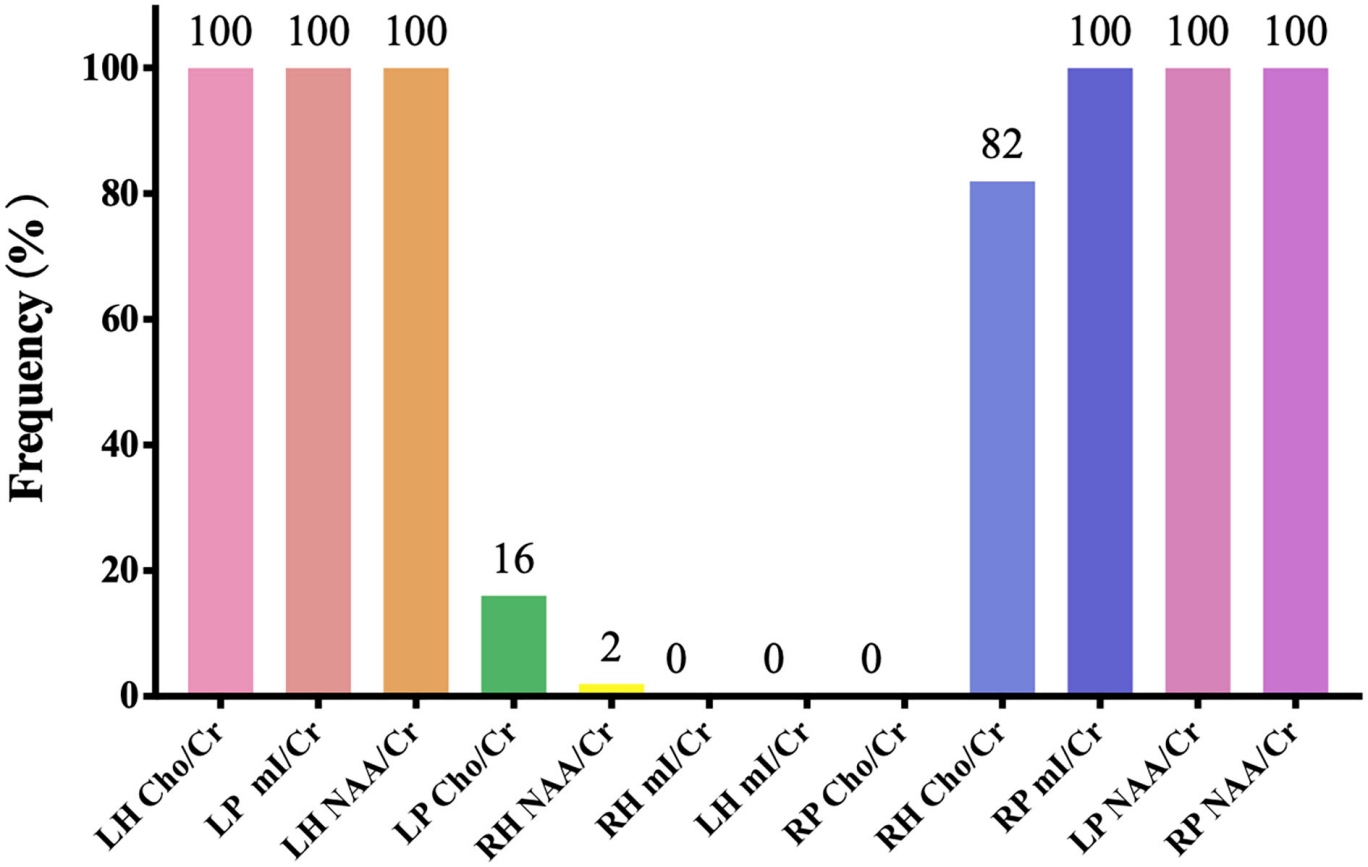
The frequency of each feature corresponding to the highest classification accuracy (LH, left hippocampus; RH, right hippocampus; LP, left prefrontal whiter matter; RP, right prefrontal whiter matter; NAA, N-acetylaspartate; Cho, choline; mI, myo-inositol; Cr, creatine).

**Figure 5:**
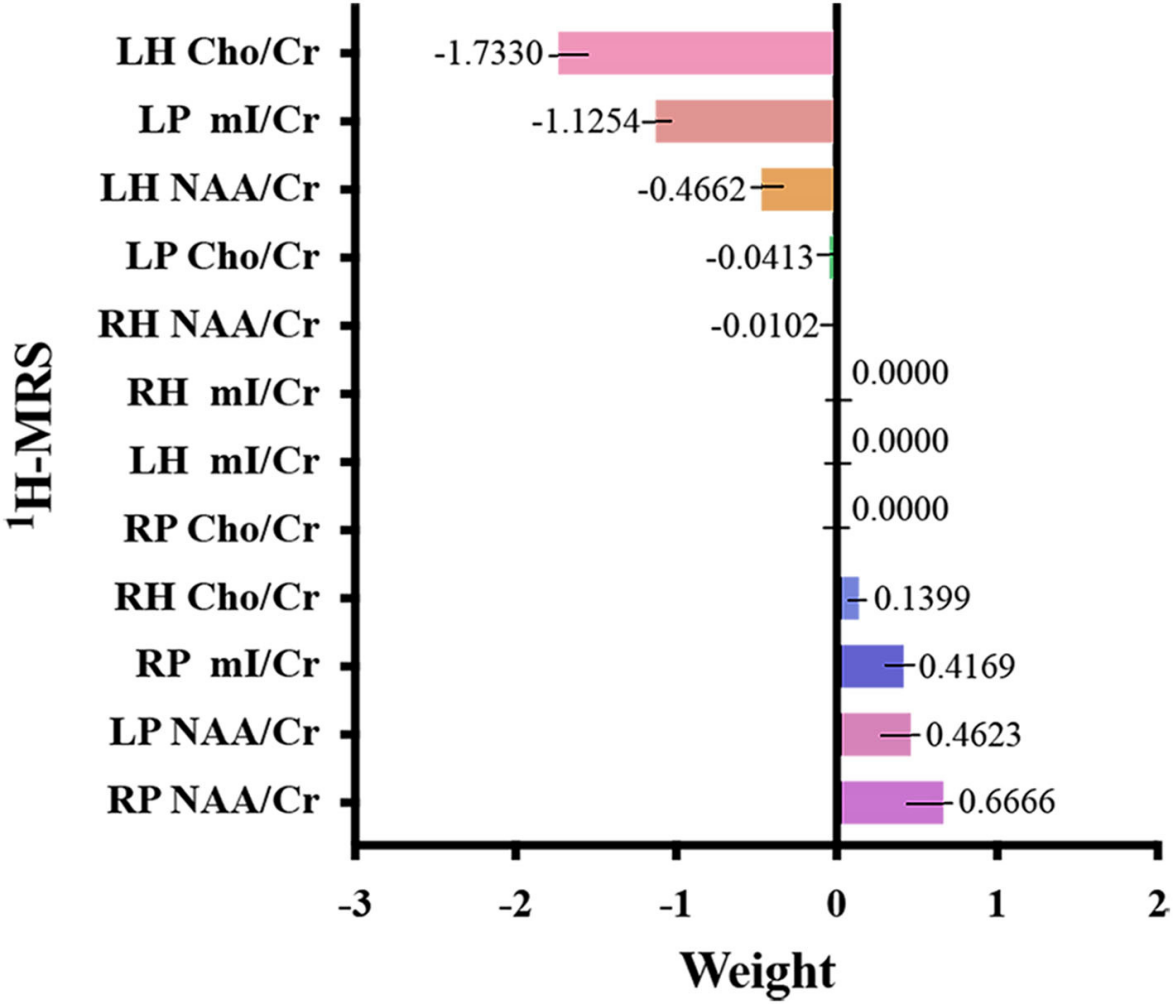
Weight value of each feature corresponding to the highest classification accuracy (LH, left hippocampus; RH, right hippocampus; LP, left prefrontal whiter matter; RP, right prefrontal whiter matter; NAA, N-acetylaspartate; Cho, choline; mI, myo-inositol; Cr, creatine).

**Figure 6:**
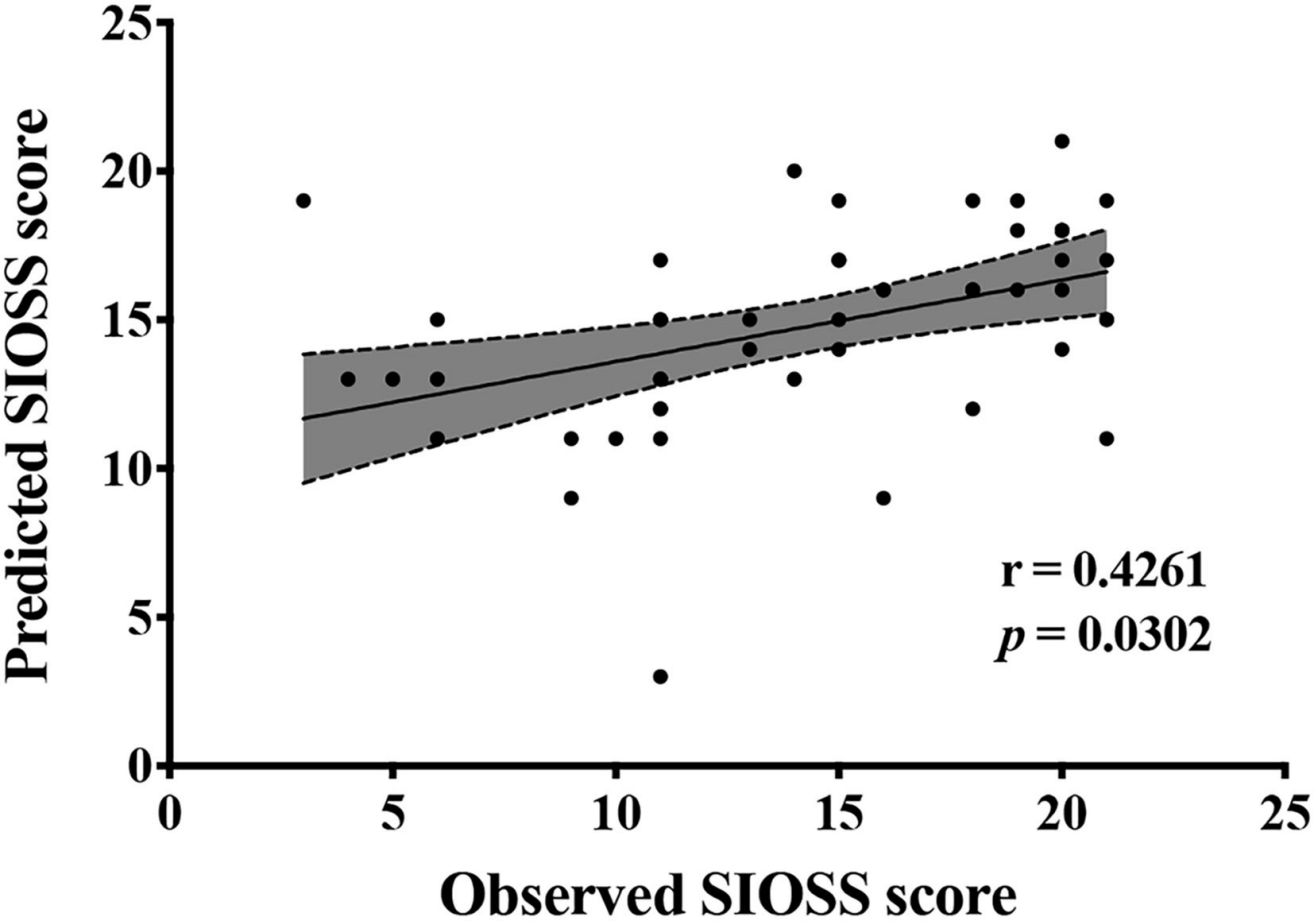
Biochemical metabolic values corresponding to the highest classification accuracy in the feature selection predict Self-rating Idea of Suicide Scale (SIOSS) scores.

Figures 2, 3, and Table 3 show the number of features, classification accuracy, sensitivity, specificity, AUC, and *p-*value obtained by the permutation test when the F-score was used for feature selection in each classifier. When the classification accuracy was 78%, the AUC was 0.77 and the number of the feature was one, the corresponding features were Cho/Cr ratios in the left hippocampus. When the classification accuracy was 72%, the AUC was 0.73 and the number of the feature was two, the corresponding features were mI/Cr ratios in the right PWM and Cho/Cr ratios in the left hippocampus. When the classification accuracy was 82%, the AUC was 0.88 and the number of the feature was three, the corresponding features were mI/Cr ratios in the bilateral PWM and Cho/Cr ratios in the left hippocampus. When the classification accuracy was 76%, the AUC was 0.85 and the number of the feature was four, the corresponding features were NAA/Cr ratios in the right PWM, mI/Cr ratios in the bilateral PWM, and Cho/Cr ratios in the left hippocampus. When the classification accuracy was 84%, the AUC was 0.90 and the number of the feature was six, the corresponding features were NAA/Cr ratios in the bilateral PWM, mI/Cr ratios in the bilateral PWM, and NAA/Cr ratios and Cho/Cr ratios in the left hippocampus. When the classification accuracy was 88%, the AUC was 0.90 and the number of the feature was seven, the corresponding features were NAA/Cr and mI/Cr ratios in the bilateral PWM, NAA/Cr and Cho/Cr ratios in the left hippocampus, and Cho/Cr ratios in the right hippocampus. When the classification accuracy was 84%, the AUC was 0.88 and the number of the feature was eight, the corresponding features were NAA/Cr and mI/Cr ratios in the bilateral PWM, Cho/Cr ratios in the left PWM, NAA/Cr ratios in the left hippocampus, and Cho/Cr ratios in the bilateral hippocampus. When the classification accuracy was 84%, the AUC was 0.85 and the number of the feature was nine, the corresponding features were NAA/Cr and mI/Cr ratios in the bilateral PWM, NAA/Cr and Cho/Cr ratios in the bilateral hippocampus, and Cho/Cr ratios in the left PWM. When the classification accuracy was 80%, the AUC was 0.85 and the number of the feature was 10, the corresponding features were NAA/Cr and mI/Cr ratios in the bilateral PWM, Cho/Cr ratios in the left PWM, NAA/Cr and Cho/Cr ratios in the bilateral hippocampus, and mI/Cr ratios in the left hippocampus. When the classification accuracy was 80%, the AUC was 0.83 and the number of the feature was 12, the corresponding features were NAA/Cr, Cho/Cr, and mI/Cr ratios in the bilateral PWM and hippocampus. According to the above results, we found that the sixth feature set (the number of the feature was seven) had the highest classification accuracy of 88% and the AUC was 0.9. Figures 4, 5 show the frequency and average weight values of each feature corresponding to the highest classification accuracy in the above feature selection process. We concluded that the NAA/Cr ratios in the bilateral PWM, the mI/Cr ratios in the right PWM, and the Cho/Cr ratios in the right hippocampus were higher and the NAA/Cr and Cho/Cr ratios in the left hippocampus and the mI/Cr ratios in the left PWM were lower in BD patients with SI than in BD patients without SI. Moreover, we found that the above seven features could be used to predict the severity of suicide risk (r = 0.4261, *p* = 0.0302) (Figure 6).

**Table 3:** Results for each classifier corresponding to feature selection.

**Feature set**	**Feature numbers**	**Sensitivity**	**Specificity**	**Accuracy (%)**	**AUC**	***p^*a*^ value***
Feature set 1	1	0.91	0.56	78	0.77	<0.001*
Feature set 2	2	0.88	0.44	72	0.73	0.277
Feature set 3	3	0.88	0.72	82	0.88	0.008*
Feature set 4	4	0.88	0.56	76	0.85	0.117
Feature set 5	6	0.94	0.67	84	0.90	0.002*
Feature set 6	7	0.94	0.78	88	0.90	<0.001*
Feature set 7	8	0.91	0.72	84	0.88	0.002*
Feature set 8	9	0.91	0.72	84	0.85	0.002*
Feature set 9	10	0.84	0.72	80	0.85	0.021*
Feature set 10	12	0.84	0.72	80	0.83	0.016*

AUC, areas under the curve; ^a^Bonferroni correction; ^*^p < 0.05 significant.

### Correlations between biochemical metabolite ratios and clinical variables in BD patients with SI

Table 4 summarizes the correlations between biochemical metabolite ratios and age of onset, duration of illness, 14-item HAMA score, and 17-item HAMD score in BD patients with SI. Notably, the NAA/Cr ratios in BD patients with SI were positively correlated with the duration of illness (r = 0.354, *p* < 0.05), and the Cho/Cr ratios in BD patients with SI were positively correlated with 17-item HAMD scores in the left hippocampus (r = 0.372, *p* < 0.05). In addition, the mI/Cr ratios in the left PWM showed a positive correlation with age of onset (r = 0.372, *p* < 0.05). However, there were no correlations between the other biochemical metabolite ratios and clinical variables in BD patients with SI (all *p* > 0.05).

**Table 4:** Correlations between biochemical metabolite ratios and clinical variables in BD patients with SI.

	**Age of onset**		**Duration of illness**		**14-item HAMA**		**17-item HAMD**	
	***r***	***p***	***r***	***p***	***r***	***p***	***r***	***p***
Left PWM								
NAA/Cr Cho/Cr mI/Cr	−0.050 −0.173 0.372	0.787 0.343 0.036*	0.084 −0.235 0.111	0.646 0.195 0.544	−0.206 0.171 −0.180	0.258 0.349 0.323	−0.335 0.042 0.037	0.061 0.819 0.841
Right PWM								
NAA/Cr Cho/Cr mI/Cr	0.024 −0.329 −0.188	0.895 0.066 0.303	0.247 −0.216 0.066	0.173 0.236 0.719	0.002 0.142 0.248	0.991 0.438 0.171	0.094 0.190 0.179	0.610 0.299 0.328
Left Hippocampus								
NAA/Cr Cho/Cr mI/Cr	−0.030 0.008 0.060	0.871 0.964 0.744	0.354 −0.318 −0.260	0.047* 0.076 0.151	0.128 0.072 0.092	0.486 0.695 0.616	0.116 0.372 0.195	0.528 0.036* 0.286
Right hippocampus								
NAA/Cr	0.108	0.555	0.050	0.787	0.126	0.492	−0.097	0.597
Cho/Cr	0.309	0.086	0.191	0.296	−0.053	0.774	−0.020	0.916
mI/Cr	0.048	0.792	−0.085	0.642	−0.146	0.424	−0.215	0.238

BD, bipolar disorder; SI, suicidal ideation; PWM, prefrontal whiter matter; NAA, N-acetyl aspartate; Cho, choline; mI, myo-inositol; Cr, creatine; HAMA, Hamilton Anxiety Scale; HAMD, Hamilton Depression Scale; ^*^p < 0.05 significant.

## Discussion

In the univariate statistical analysis, we found that the Cho/Cr values in the left hippocampus of BD patients with SI were decreased, suggesting that BD patients with SI had a decrease in membrane phospholipid metabolism in the hippocampus. In addition, the AUC of Cho/Cr values in the left hippocampus was relatively high (0.79), indicating that it has good diagnostic accuracy for BD patients with SI. To the best of our knowledge, there have been no studies on ^1^H-MRS in the hippocampus of BD patients with SI. Previous studies have suggested that functional disorders of the frontal limbic network play an essential role in the mechanisms associated with suicide among patients with BD (Giakoumatos et al., 2013). The frontal limbic network is composed of the frontal lobes, cingulate gyrus, and subcortical brain structures, such as the hippocampus, amygdala, and other brain regions (Phillips et al., 2003, 2008). Among them, the hippocampus has excellent effects on encoding and recalling the emotional meaning of events, which may affect the emotional response and regulation process (Richard-Devantoy et al., 2015). Studies have indicated that the gray matter volume in the hippocampus and orbitofrontal cortex of BD patients with suicide attempts, compared with BD patients without suicide attempts, was significantly decreased (Johnston et al., 2017), and the amplitude of ALFF values in the right hippocampus was increased in severely depressed patients with a history of suicide attempts (Lan et al., 2019). Thus, the hippocampus also plays a major role in the mechanism of suicide in patients with BD. Cho is one of the components of membrane phospholipid metabolism that is closely related to membrane phospholipid decomposition and synthesis, reflects the metabolic level of membrane phospholipids, and is a marker of membrane integrity (Strakowski et al., 2005). In addition, Cho is more abundant in astrocytes and oligodendrocytes, which reflects changes in glial metabolism and function (Atmaca and Yildirim, 2012). A recent study found that cerebral vimentin-immunoreactive astrocytes showed a widespread reduction in depressive disorder patients who died of suicide, suggesting that dysfunction in astrocytes was associated with suicide and depression (O'Leary et al., 2021), and the results of our study provide new evidence for this.

In the MVPA, our results found that the highest classification accuracy obtained by putting all features into the classifier was 88% and the AUC was 0.90, which was higher than the diagnostic prediction ability of the Cho/Cr values in the left hippocampus in univariate statistical analysis (AUC = 0.79). This suggested that the use of multiple biochemical metabolic values more effectively identified SI in patients with BD. Based on the number of features, the frequency of feature occurrence, and the weight values obtained by feature selection, our results suggested that in addition to the metabolic values identified in the univariate statistical analysis, we further found that the NAA/Cr values in the bilateral PWM, the mI/Cr values in the right PWM, and the Cho/Cr values in the right hippocampus were higher in BD patients with SI than in those without SI and the NAA/Cr values in the left hippocampus and the mI/Cr values in the left PWM were lower in those with SI than in those without SI by using the MVPA. Many neuroimaging studies have reported that the dysfunction of some neural circuits is related to SI and behavior (Ding et al., 2017; Johnston et al., 2017; Brown et al., 2020). The abnormal function of the frontal lobes could lead to disorders of emotional executive control and emotional pain processing (van Heeringen et al., 2010). The results of some studies have shown that dysfunction in the PWM regions was most common in BD patients with a history of suicide attempts (Hozer and Houenou, 2016). BD patients with a history of suicide attempts had decreased white matter in the caudal frontal lobe and the left orbitofrontal cortex (Mahon et al., 2012; Johnston et al., 2017). Therefore, abnormal white matter in the prefrontal lobes may be related to the pathogenesis of suicide in patients with BD. NAA reflects the vitality of neurons and can maintain the osmotic pressure of neurons cell (Baslow, 2003). NAA content is closely related to the function of neurons and is a marker of neuron density and survival. Simultaneously, NAA is related to the function of mitochondria and myelin formation and is an energy marker of neuronal mitochondria. The results of our study suggested that BD patients with SI had enhanced prefrontal neuronal function or mitochondrial dysfunction. At present, there are many controversies about the study of NAA in the prefrontal lobes of patients with BD. Some studies suggested that the NAA values in the prefrontal lobes were decreased, while others thought that the NAA values were increased or at a normal level (Zhong et al., 2014, 2018; Moon et al., 2015; Liu et al., 2017b; Patel et al., 2018). The reasons for the inconsistent results may be related to magnetic resonance technical parameters, disease status, the influence of drugs, and other factors. For example, studies have reported that NAA values were increased in the prefrontal cortex after lithium treatment (Hajek et al., 2012). A follow-up study of BD patients treated with lithium for 4 weeks also confirmed that lithium could induce an increase in NAA levels (Moore et al., 2000). More high-quality samples need to be evaluated for further discussion in future.

Myo-inositol exists in glial cells and is a marker of glial cells. It is involved in the circulation of inositol phosphate, the regulation of neuronal permeability, and the catabolism of phospholipids in the cell membrane. It has a nutritional and protective effect on neurons (Dager et al., 2008). In the MVPA, our study found that the mI/Cr values in the right PWM were increased and the mI/Cr values in the left prefrontal matter were decreased in BD patients with SI. According to previous studies, we learned that when the synthesis of inositol phosphate is blocked, mI content will increase, and the excitatory transmitter induced by inositol phosphate will decrease, thus resulting in depression (Wu et al., 2001). In addition, a plasma metabolomic study found that the peak of inositol was higher in depressive disorder patients with suicide attempts than in depressive disorder patients without suicide attempts (Zhou, 2013). Based on the above results, it could be concluded that the increase in inositol in depressed patients with BD might be related to suicide. However, there were also inconsistent findings. Shimon et al. (1997) measured the content of the inositol and its synthetic enzyme, inositol monophosphatase in postmortem brain samples of suicide victims, patients with BD, and normal controls. The results showed that the levels of inositol in the frontal cortex of the suicide victims and patients with BD were significantly lower than those of the normal controls. We found that the changes in inositol levels in the left and right PWM were inconsistent in BD patients with SI, which might be related to the asymmetry of frontal lobe function. Therefore, our results indicated that the abnormal level of inositol metabolism or the disorder of glial cell function in the PWM might be the pathophysiological mechanism in BD patients with SI.

The above research results showed that, first, the metabolic values with differences in univariate statistical analysis coincided with the results of the MVPA, and the frequency of the occurrence of the feature included in the highest classification accuracy of 88% was consistent with the size of features average weight values, indicating that our model has good stability and high feature sensitivity. Second, the results of this study also fully confirmed the advantage of MVPA, i.e., it could find brain biochemical metabolic differences that could not be detected by univariate statistical analyses. Through the MVPA results, we found that the neuron function in the PWM and hippocampus, inositol metabolism level in the PWM, and membrane phospholipid catabolism level in the hippocampus were functionally disrupted. These differences in brain biochemical metabolism values might also be neurobiological mechanisms associated with BD with SI. Moreover, we further found that the biochemical metabolic values corresponding to the highest classification accuracy in the feature selection could predict SIOSS scores in patients with BD. At present, many studies have focused on univariate statistical analyses to explore the correlations between imaging characteristics and clinical variables in BD patients with SI. A few studies have recently used the multiple regression model and LOOCV method to predict suicide scale scores with dynamic ALFF values in BD and depression patients with SI (Li et al., 2019; Gong et al., 2020). However, we are not aware of any study that has reported using biochemical metabolic values to predict the severity of SI based on MVPA. In the current study, we found that biochemical metabolic values could successfully predict the severity of SI in patients with BD, which further suggested that biochemical metabolic values may be a more powerful predictive neural marker for SI among patients with BD. In summary, according to our research results, we firmly believe that the combination of multiple biochemical metabolites can help to identify BD patients with SI and predict the severity of SI, thereby reducing suicide mortality, and the model had sufficiently high enough accuracy, sensitivity, and specificity.

In the correlation analysis between brain biochemical metabolites and clinical variables in BD patients with SI, we found that the NAA/Cr values in the left hippocampus were positively correlated with the duration of illness, suggesting that the longer the duration of illness was, the more active the neuronal function in the left hippocampus among BD patients with SI. Some studies have shown that suicide attempts by patients with BD are related to longer duration of illness (Lijffijt et al., 2014). The NAA/Cr values in the hippocampus of patients with BD I were positively correlated with the duration of illness (Cui et al., 2009), which was consistent with the results of our study. However, some studies have found that the hippocampal NAA/Cr values in first-episode patients with BD are not associated with the duration of illness (Atmaca et al., 2006). The inconsistencies in the results of the above studies might be related to the selected samples and the influence of medications. In future, first-episode patients with BD need to be selected for further evaluation. Moreover, our study also found that the mI/Cr values in the left PWM were positively correlated with the age of onset. Some studies have suggested that there was a significant correlation between suicidal behavior and age of onset in patients with BD (Song et al., 2012). Singh et al. (2013) showed that mI/Cr values were related to age and increased with age. Therefore, our results suggested that the level of inositol metabolism in the left PWM of BD patients with SI may increase with the age of onset. In addition, our results showed that the Cho/Cr values in the left hippocampus were positively correlated with HAMD scores, suggesting that the more severe the degree of depression in BD patients with SI was, the stronger the level of membrane phospholipid catabolism in the left hippocampus.

To the best of our knowledge, this study is the first to explore the differences in brain biochemical metabolites in BD patients with SI and use abnormal metabolite ratios to predict the severity of suicide risk based on the SVM algorithm. However, our research also has some limitations. First, our sample size was limited, and there were no data from multiple centers to verify each other, so the generalization ability of the model needs to be further confirmed. Second, the samples were unbalanced, which may affect the robustness of the model. In future, we need to expand the sample size and maintain the sample balance for further research and discussion. In addition, this study focused only on ^1^H-MRS, which could be combined with other imaging techniques to find high-accuracy suicide predictors for patients with BD at multiple levels. Finally, this study was a cross-sectional study, which was unable to predict whether BD patients with SI will commit suicide, and therefore, longitudinal research will be needed to further elaborate on the future suicide risk of patients with BD.

## Conclusion

In conclusion, our study showed that the combination of multiple brain biochemical metabolites could better predict the risk and severity of suicide in patients with BD. Moreover, the abnormal levels of inositol metabolism in the PWM, neuron function in the PWM and hippocampus, and membrane phospholipid catabolism level in the hippocampus may be neuropathophysiological mechanisms underlying SI among patients with BD. In addition, in BD patients with SI, the level of neuronal function in the left hippocampus may be related to the duration of illness, the level of membrane phospholipid catabolism in the left hippocampus may be related to the severity of depression, and the level of inositol metabolism in the left PWM may be related to the age of onset. In future, we can expand the sample size and conduct multicenter prospective research combined with multimodal data, including genetics, electroencephalography, and different imaging methods, to further elucidate suicide prediction indicators in patients with BD to provide a basis for the early detection of suicide behavior in patients with BD.

## Data availability statement

The data used to support the findings of this study are available from the corresponding author upon request.

## Ethics statement

The studies involving human participants were reviewed and approved by Ethics Committee of the People's Hospital of Xinjiang Uygur Autonomous Region. The patients/participants provided their written informed consent to participate in this study. Written informed consent was obtained from the individual(s) for the publication of any potentially identifiable images or data included in this article.

## Author contributions

JC, XZ, CZ, SZ, and HT conceived and designed this study. JC, XZ, YP, and YS conducted the data analysis. YQ performed the magnetic resonance imaging scanning. JC and SZ collected the data. JC, CZ, SZ, and HT wrote and critically reviewed the first draft of the manuscript. All authors contributed to the writing, critical review, and approval of the final manuscript.

## Funding

This work was supported by the National Key Research and Development Program of China (2018YFC1314301), the Natural Science Foundation of Xinjiang Uygur Autonomous Region (2017D01C109), and the Tianshan innovation team project of Xinjiang Uygur Autonomous Region (2022D14011).

## Conflict of interest

The authors declare that the research was conducted in the absence of any commercial or financial relationships that could be construed as a potential conflict of interest.

## Publisher's note

All claims expressed in this article are solely those of the authors and do not necessarily represent those of their affiliated organizations, or those of the publisher, the editors and the reviewers. Any product that may be evaluated in this article, or claim that may be made by its manufacturer, is not guaranteed or endorsed by the publisher.
